# Midazolam Ameliorates the Behavior Deficits of a Rat Posttraumatic Stress Disorder Model through Dual 18 kDa Translocator Protein and Central Benzodiazepine Receptor and Neurosteroidogenesis

**DOI:** 10.1371/journal.pone.0101450

**Published:** 2014-07-02

**Authors:** Yu-Liang Miao, Wen-Zhi Guo, Wen-Zhu Shi, Wei-Wu Fang, Yan Liu, Ji Liu, Bao-Wei Li, Wei Wu, Yun-Feng Li

**Affiliations:** 1 Department of Anesthesiology, Chinese PLA No. 306 Hospital, Beijing, P.R. China; 2 Department of Anesthesiology, Beijing Military General Hospital of the PLA, Beijing, P.R. China; 3 Anesthesia and Operation Center, Hainan Branch of Chinese PLA General Hospital, Sanya, P.R. China; 4 Department of Head and Neck Surgery of Otolaryngology, Chinese PLA No. 306 Hospital, Beijing, P.R. China; 5 Institute of Pharmacology and Toxicology, Academy of Military Medical Sciences, Beijing, P.R. China; Max Planck Institute of Psychiatry, Germany

## Abstract

Post-traumatic stress disorder (PTSD) is a debilitating anxiety disorder that may develop after an individual has experienced or witnessed a severe traumatic event. It has been shown that the 18 kDa translocator protein (TSPO) may be correlated with PTSD and that the TSPO ligand improved the behavioral deficits in a mouse model of PTSD. Midazolam, a ligand for TSPO and central benzodiazepine receptor (CBR), induces anxiolytic- and anti-depressant-like effects in animal models. The present study aimed to determine whether midazolam ameliorates PTSD behavior in rats as assessed by the single prolonged stress (SPS) model. The SPS rats received daily Sertraline (Ser) (15 mg/kg, p.o.) and midazolam (0.125, 0.25, 0.5, and 1 mg/kg, p.o.) during the exposure to SPS and behavioral assessments, which included the open field (OF) test, the contextual fear paradigm (CFP), and the elevated plus-maze (EPM). The results showed that, like Ser (15 mg/kg, p.o.), midazolam (0.25 and 0.5 mg/kg, p.o.) significantly reversed the behavioral deficiencies of the SPS rats, including PTSD-associated freezing and anxiety-like behavior but not the effects on spontaneous locomotor activity. In addition, the anti-PTSD effects of midazolam (0.5 mg/kg, p.o.) were antagonized by the TSPO antagonist PK11195 (3 mg/kg, i.p.), the CBR antagonist flumazenil (15 mg/kg, p.o.) and the inhibitor of steroidogenic enzymes finasteride (30 mg/kg, p.o.), which by themselves had no effect on PTSD-associated freezing and anxiety-like behavior. In summary, this study demonstrated that midazolam improves the behavioral deficits in the SPS model through dual TSPO and CBR and neurosteroidogenesis.

## Introduction

Post-traumatic stress disorder (PTSD) can develop following exposure to severe traumatic events, such as the events that occur during war, after a violent personal assault, or following natural disasters [1]. A majority of PTSD patients exhibit long-lasting re-experience of traumatic events and subsequently avoid the stimuli that link traumatic events, even though they recognize that the traumatic event is no longer occurring. Meanwhile, broad deterioration and impairments of cognitive function have also been reported in PTSD patients [2]. Sufferers continue to re-experience trauma-related anxiety and distress even after the cessation of the trauma [3]. The dysregulation of several biological systems have been implicated in the abnormal response underlying the pathophysiology of PTSD, a complex disorder, and the affected biological pathways may include corticotrophin releasing hormone (CRH) [4], hypothalamic-pituitary-adrenal (HPA) axis abnormalities [5], and dysfunction in the noradrenergic [6], serotonergic [7], [8], and glutamatergic systems [9].

As a general principal, the selective serotonin reuptake inhibitors (SSRIs), such as sertraline and paroxetine, are a first-line treatment option for the core PTSD symptoms [10], [11]. However, SSRIs have large disadvantages, such as a delayed onset of action, partial response with residual symptoms or non-responsiveness, and severe side effects [12]. Based on these disadvantages, future work needs to search for novel pharmacological targets to provide the next generation of anti-PTSD drugs.

The 18 kDa translocator protein (TSPO), a promising target for treating neurological disorders without benzodiazepine-like side effects [12], [13], may be associated with PTSD [14]. The TSPO, which is located mainly in the outer mitochondrial membrane (OMM) in peripheral tissues and the central nervous system (CNS), processes the availability of neurosteroids in the brain by modulating cholesterol [15]. Several studies have shown that TSPO ligands effectively treated depression and anxiety disorders through the modulation of the neurosteroid synthesis [16], [17]. Midazolam, a ligand for TSPO and CBR, has also induced anxiolytic-like and antidepressant effects [18], [19]. These observations have led to the hypothesis that midazolam may also be effective in improving stress-induced psychiatric conditions, including PTSD.

The physiological and behavioral changes observed in animals exposed to the single prolonged stress (SPS) model can represent the pathophysiological process and core symptomatology of PTSD, including anxiety behavior and cognitive impairments [20], [21]. SPS paradigms have been extensively utilized in the investigation of anxiety disorders, especially PTSD [20]. A rat model involving SPS has been developed and employed for PTSD research [22]. Because the behavioral responses observed in rats subjected to SPS resemble the clinical symptoms of PTSD patients, the SPS paradigm has been postulated as an appropriate animal model of PTSD.

Currently, no reports have examined the therapeutic effects of midazolam on PTSD in pre-clinical animal model experiments. Midazolam's direct interact to CBR and high affinity for TSPO lead to the anxiolytic-like effects and induce the hypothesis [23] that the anti-PTSD effects of midazolam might be mediated by the CBR and TSPO. The present study was conducted to investigate the anti-PTSD effects of midazolam and the underlying mechanism.

## Materials and Methods

### 1 Animals and housing

Male Sprague-Dawley rats were obtained from the Beijing SPF Animal Technology Company (Beijing, China). These animals were selected because Sprague-Dawley rats exhibit PTSD-like symptoms [21]. The animals were housed in a temperature- and humidity-controlled room. All the animals were housed with a 12-h light/dark cycle starting at least 5 days before the experiment and had access to water and food ad libitum. They were group-housed with the same cage-mates throughout the acclimation and testing periods and randomly assigned to the experimental groups (10 animals each). The animal experiments were approved by the animal ethical committee of the academy of military medical sciences. All experiments performed were in compliance with current laws and regulations and adhered to the National Institutes of Health Guide for the Care and Use of Laboratory Animals (NIH publication No. 86-23, revised 1996).

### 2 Drugs and drug administration

**Figure 1:**
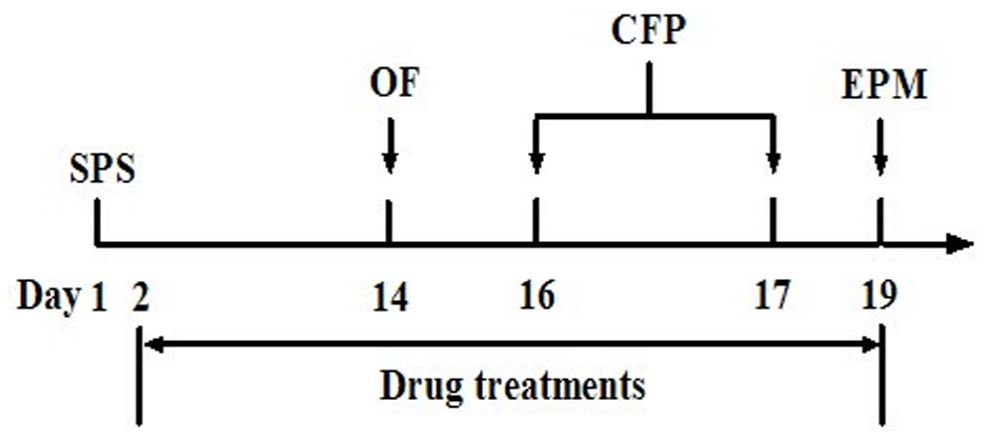
Treatment and behavioral tests schedules for the SPS model of PTSD. Animals were subjected to SPS on day 1. From days 14 to 19, animals were subjected to testing sessions composed of 3 different behavioral tests: OF, Open field; CFP, Contextual fear paradigm; and EPM, Elevated plus maze. Ser and midazolam were administered daily during the SPS exposure and behavioral assessments from days 2 to 19. PK11195, flumazenil or finasteride were administered 30 min before behavioral testing, respectively.

Based on previous studies, Sertraline (Ser) (St Louis, MO, U.S.A.) was administered at a dose of 15 mg/kg (p.o.) as a positive control in the behavioral tests [17], [23]. Midazolam, flumazenil and finasteride was purchased from Sigma-Aldrich. Ser, midazolam, flumazenil and finasteride were dissolved in saline. PK11195 (St Louis, MO, U.S.A.) was suspended for injection (i.p.) in saline containing 2% DMSO and 0.8% Tween 80 [24]. The compounds were administered by gavage at a dose of 2 mL/kg. The drug administration regimen was based on the treatment regimen for PTSD animal models with minor modifications [23]. Ser (15 mg/kg, p.o.) or midazolam (0.125, 0.25, 0.5, and 1 mg/kg, p.o.) was given once daily. The doses of PK11195 (1 and 3 mg/kg, i.p.), flumazenil (5 and 15 mg/kg, p.o.) and finasteride (10 and 30 mg/kg, p.o.) were based on the published literatures with minor modifications [2], [25]–[28]. All of these three antagonists were administered 30 min before testing, respectively. The design of the treatment schedule and behavioral tests is outlined in Fig. 1.

### 3 The establishment of the SPS model

The SPS model was performed as previously described with minor modifications [29]. SPS was conducted in three stages: restraint (2 h), forced swim (20 min), and ether anesthesia. Each rat was restrained for 2 h by placing it inside a disposable clear polyethylene cone bag (Asahikasei, Tokyo, Japan) with only the tail protruding [30]. The large end of the cone was closed with tape at the base of the tail. The bag size was adjusted according to the size of the rat to achieve complete immobilization. A hole in the small end of the cone allowed the rats to breathe freely. After immobilization, they were individually placed in a clear acrylic cylinder (240 mm diameter, 500 mm height), filled two-thirds with water (24 °C), and forced to swim for 20 min. Following 15 min of recuperation, they were exposed to diethyl ether anesthesia until loss of consciousness and then left in their home cage. Different reagents were administered daily from days 2 to 19. Behavioral experiments were performed at days 14, 16, 17 and 19, respectively (Fig. 1).

### 4 Long term effects of midazolam on the behavior of SPS-exposed rats

#### 4.1 Open field (OF) test

We investigated whether the SPS model and midazolam treatment affected locomotor activity. The OF test was performed as previously described with minor modifications [31], [32]. Rats were placed in the corner of a plastic box (76×76×46 cm) with the base divided into equal squares for a 5-min acclimation period. Subsequently, the number of crossings (all 4 paws placed into a new square), rears (both front paws raised from the floor), and fecal pellets were automatically recorded by the VIDEO-MEX-V image analytic system (Columbus Instruments, USA) in the subsequent 5 min. The OF test and the following experiments were performed in a room with dim light.

#### 4.2 Contextual fear paradigm (CFP)

The CFP test is based on previous studies that showed a freezing response upon re-exposure to the traumatic context can be used as a measure of conditioned associative fear memory, which reflects the response to trauma-related cues, a symptom of PTSD [23], [33]. CFP was performed as previously described with minor modifications [23]. Each rat was exposed to a 180-s conditioning context without stimulation. Immediately after, a foot shock (0.8 mA, 4 s) through a stainless steel grid floor (Med Associates Inc., USA) was administered. Following the foot shock, the rats remained in the chamber for an additional 1 min before they returned to their home cages. Each rat was placed in the conditioning chamber, where it had previously received a foot shock, 24 h after the initial foot shock, and the contextual fear response was evaluated by measuring the duration of freezing behavior during a 5-min interval. Freezing was defined as the absence of all movement except those movements necessary for respiration [34]. Motion thresholds (18) and number of frames (30) were selected by the VideoFreeze system (with IEEE 1394 digital video; 8-bit pixel per mm2 @ 30 Hz; Med Associates, INC, USA), and were corroborated by the observers [17], [35]. Total cumulative freezing time (total seconds of freezing during each assessment period) was measured and analyzed automatically by the computer software (Med Associates Inc. Video Freeze SOF-843., USA). The percentages of freezing time were calculated by dividing the freezing time by the total time [36].

#### 4.3 Elevated plus maze (EPM)

The EPM is one of the most widely used assessments to evaluate the PTSD-associated anxiogenic-like behavior in rodents [23], [36], [37]. The EPM has been well validated in detecting responses to external stressful stimuli (Liebsch et al., 1998). The rat was placed in the center of the maze (60 cm above the floor) facing an enclosed arm. The time spent in and number of entries into (with all 4 paws) both the open and enclosed arms (60×12 cm) were recorded for 5 min as previously described [37]. In this test, open-arm activity was defined as the time stayed in the open arms relative to the total time spent in open and closed arms (open/total×100), and the number of entries into open arms relative to the total number of entries into both arms (open/total×100) [36]. The tracking software “Viewer 3.0 (Biobserve)” was used to recorded and analyzed the behavior activity. The percentages of time spent in and the entries into the open arms were calculated by dividing the time spent in and the entries into the open arms by the total time spent in and the total arm entries into both arms, respectively.

### 5 Statistical analysis

Unless otherwise specified, statistical analysis was performed by GraphPad Prism 5.0 (GraphPad Software Inc., San Diego, CA). All data are presented as the mean±S.E.M. The statistical significance of experimental observations was determined by one-way analysis of variance (ANOVA) or two-way ANOVA followed by Bonferroni's multiple comparison tests, as indicated in the results section. Differences with *p*<0.05 were considered significant for all the tests.

## Results

### 1 Effects of midazolam on the locomotor activity of rats

**Figure 2:**
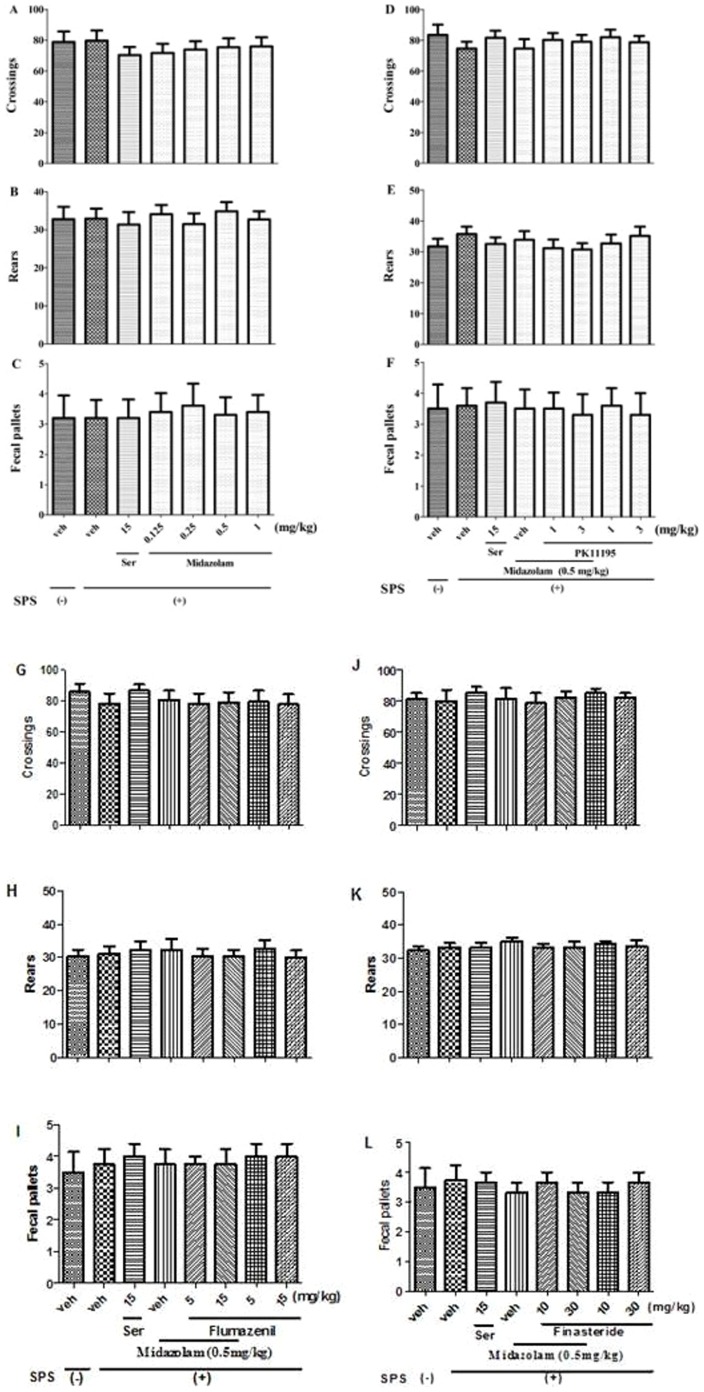
Effects of midazolam on the locomotor activity of rats. None of the treatments altered the number of line crossings (A, D, G and J), rears (B, E, H and K), and fecal pallets (C, F, I and L) in the OF test (n = 10).

The effects of repeated administration of midazolam on locomotor activity are shown in Fig. 2. There was no significant effect on the number of line crossings (*p*>0.05, Fig. 2A, 2D, 2G and 2J), rears (*p*>0.05, Fig. 2B, 2E, 2H and 2K), or fecal pellets (*p*>0.05, Fig. 2C, 2F, 2I and 2L) between the groups. These results indicated that none of the repeated reagents treatment affected the locomotor activity of rats.

### 2 Effects of midazolam on the contextual freezing of rats

**Figure 3:**
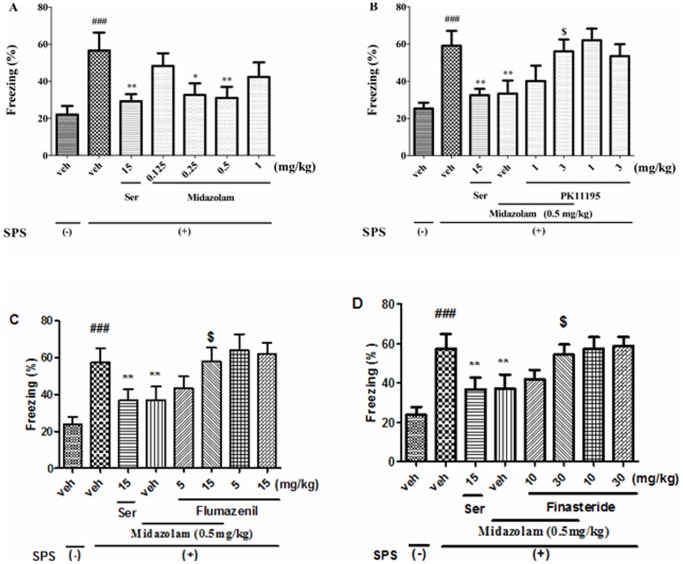
Effects of midazolam on the freezing behavior of rats subjected to SPS. The freezing time was reduced by midazolam (0.25 and 0.5 mg/kg, p.o.) (A), which was reversed by PK11195 (3 mg/kg, i.p.) (B), flumazenil (15 mg/kg, p.o.) (C), or finasteride (30 mg/kg, p.o.) (D). ^###^ *p*<0.001 vs. vehicle-treated SPS (-) group; ^*^ *p*<0.05, ^**^ *p*<0.01 vs. vehicle-treated foot shock (+) group; ^$^ *p*<0.01 vs. midazolam-treated group (0.5 mg/kg, p.o.) (n = 10).

The effects of midazolam on the contextual freezing behavior of the rats are shown in Fig. 3. After the exposure to SPS, the freezing time was significantly increased in rats, which was significantly reversed by Ser (15 mg/kg, p.o.) or midazolam (0.25 and 0.5 mg/kg, p.o.) (*p*<0.01, Fig. 3A). In addition, midazolam at a dose of 0.5 mg/kg (p.o.), which was the dose that induced the maximal reduction in the freezing time, was antagonized by PK11195 (3 mg/kg, i.p., *p*<0.01, Fig. 3B), flumazenil (15 mg/kg, p.o., *p*<0.01, Fig. 3C) or finasteride (30 mg/kg, p.o., *p*<0.01, Fig. 3D). These results indicated that repeated midazolam treatment alleviated contextual freezing behavior in these rats, which was mediated by TSPO and CBR and neurosteroidogenesis.

### 3 Effects of midazolam on rat EPM performance

**Figure 4:**
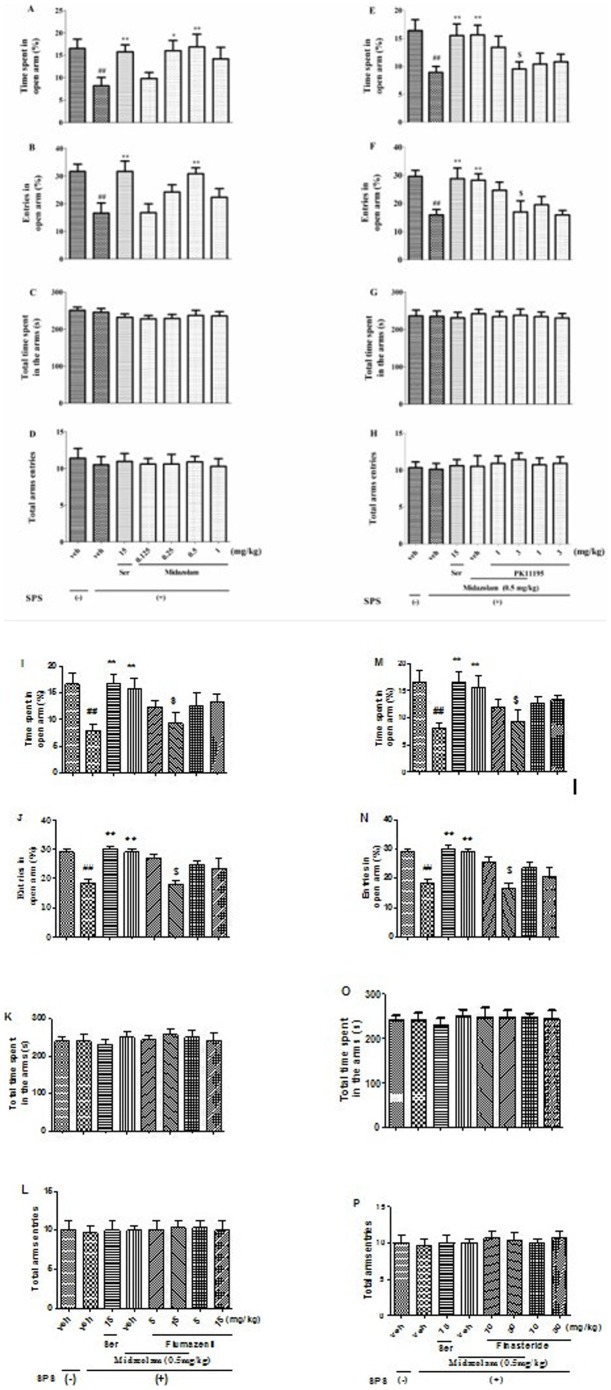
The anxiolytic-like effects of repeated midazolam treatment and the antagonistic effects of PK11195, flumezenil or finasteride in SPS-exposed rats as measured by percentages of time spent in (A, E, I, M) and entries into (B, F, J, N) the open arms of the EPM, total time spent in the arms (C, G, K, O), and total arm entries (D, H, L, P), respectively. ^##^ *p*<0.01 vs. vehicle-treated foot shock (−) group; ^*^ *p*<0.05, ^**^ *p*<0.01 vs. vehicle-treated foot shock (+) group; ^$^ *p*<0.05 vs. midazolam-treated group (0.5 mg/kg, p.o.) (n = 10).

As shown in Fig. 4, the percentage of the time spent in and the entries into the open arms of the EPM was significantly reduced in SPS-exposed rats, but repeated administration of Ser (15 mg/kg, p.o.) or midazolam significantly increased the percentage of the time spent in (0.25 and 0.5 mg/kg, p.o.) (*p*<0.05; Fig. 4A) and the entries (0.5 mg/kg, p.o.) (*p*<0.01; Fig. 4B) into the open arms. In addition, there was no significant difference in the total time spent in (*p*>0.05; Fig. 4C) and total arm entries (*p*>0.05; Fig. 4D) into the arms of the EPM between the groups. Meanwhile, 0.5 mg/kg of midazolam, which induced the maximal increase in the percentage of the time spent in (*p*<0.01; Fig. 4E, 4I and 4M) and entries (*p*<0.01; Fig. 4F, 4J and 4N) into the open arms, was antagonized by PK11195 (3 mg/kg, i.p.), flumazenil (15 mg/kg, p.o.) or finasteride (30 mg/kg, p.o.) respectively, without affecting the total time spent in (*p*>0.05; Fig. 4G, 4K and 4O) and total entries (*p*>0.05; Fig. 4H, 4L and 4P) into the EPM arms. These results indicated that exposure to SPS elicited PTSD-associated anxiogenic-like behavior in rats and that similar to Ser, midazolam ameliorated PTSD-associated anxiogenic-like behavior. These effects were mediated by TSPO, CBR and neurosteroidogenesis.

## Discussion

The present study was performed to investigate the possible mechanism of the anti-PTSD effects of repeated midazolam treatment using the SPS model. Similar to Ser, midazolam significantly ameliorated the PTSD-associated freezing and anxiety-like behaviors without affecting spontaneous locomotor activity, and these effects were antagonized by PK11195, flumazenil or finasteride. We found that the anti-PTSD effects of midazolam were mediated by TSPO, CBR and neurosteroidogenesis.

Furthermore, the results in the present study indicate that SPS-induced freezing and anxiety, which is consistent with previous studies showing that the SPS model produces enhanced contextual freezing and increases anxiety-like behavior in the elevated plus maze (EPM) test and stress-induced analgesia compared with rats subjected to a sham treatment [21], [23], [38]. In addition, these results are also supported by other animal models with similar behavioral deficits [17], [36]. The SPS-induced freezing behavior is also supported by studies showing that PTSD is characterized by an exaggerated reaction to a mild stressor or the reminder of the trauma to which the response is appropriate for the original traumatic situation. Moreover, the decrease in open arm exploration of the EPM after SPS-exposure resembled the observation in PTSD patients who showed enhanced anxiety and fear in response to stimuli unrelated to trauma [3]. The underlying mechanism may be that SPS elicited the acquisition of conditioned fear responses to trauma-related and trauma-unrelated stimuli [23]. The behavioral deficits of PTSD might be associated with the prefrontal cortex, hippocampal dysfunction, and structural abnormalities [23], which are the brain regions that play an important role in the process of fear conditioning, emotional processing, and explicit memory. Additionally, consistent with a previous study [23], the SPS model induced behavioral deficits without effects on the locomotor activity, suggesting that freezing and PTSD-associated anxiogenic-like behavior may be not generated through changes in locomotor activity that correlate with physiological changes. The data presented in this study support the idea that this PTSD model resembles some of the clinical manifestations of PTSD.

We also found that Ser improved the behavioral deficits of this SPS model, which is consistent with a previous study [23]. Similar to Ser, midazolam reduced the freezing in the CFP test and increased open arm exploration in the EPM, which indicated that midazolam significantly ameliorated the contextual fear and PTSD-associated anxiogenic-like behavior in rats. The range of the doses was similar to previous studies examining the anxiolytic-like effects of midazolam [39], [40]. Unlike other benzodiazepines such as diazepam and clonazepam only weakly interacting with TSPO [25], [26], midazolam has high affinity to TSPO. In the present study, the anti-PTSD effects induced by midazolam were mediated at least partially by TSPO, which is evidenced by the antagonism of the anxiolytic-like effects of PK11195 and the lack of significant effects on freezing and PTSD-associated anxiogenic-like behavior. Similar to the anxiolytic-like effects of other TSPO ligands, such as AC-5216, our results were consistent with a previous study that showed the anxiolytic-like effects of AC-5216 were antagonized by PK11195, which itself had no significant effect on the number of shocks in the Vogel-type conflict test [24]. Moreover, the findings that the anti-PTSD effects of midazolam are not mediated by a change in locomotor activity supported the anti-PTSD- [17], anxiolytic- [22], [26], and anti-panic effects [41] of AC-5216. The lack of effects on spontaneous locomotor activity might be associated with the selective affinity of midazolam for TSPO [42], and the dose of midazolam applied in our study was insufficient for the effective sedation, in turn inhibiting the spontaneous locomotor activity.

The anti-PTSD effects of midazolam might also be associated with the neurosteroidogenesis of allopregnanolone, which is known to interact with GABA_A_ receptor through a neurosteroid recognition site directly and different to CBR [43], [44]. These anti-PTSD effects were similar to selective brain steroidogenic stimulants (SBSSs), which rapidly increased corticolimbic allopregnanolone levels and normalized the exaggerated contextual fear responses [45]. Our results agree with many other studies that have observed the anxiolytic and antidepressant effects elicited by allopregnanolone. The reduction of allopregnanolone on tonic GABA-mediated inhibitory conductance might contribute to the pathophysiology of aggression, anxiety, and PTSD [46], [47]. The potential mechanism might be that the decrease in extrasynaptic GABA_A_ receptor-mediated tonic inhibition facilitates phasic increases in glutamatergic and GABAergic activity, which could disrupt the frontal lobe-mediated inhibition of the amygdale [48], might contribute to an enhancement of fear conditioning and resistance to extinction of fearful conditioned responses, and promote the development of PTSD [47]. Midazolam might be associated with the biosynthesis of allopregnanolone. Previous studies have shown that administering allopregnanolone doses that normalized hippocampal allopregnanolone levels reversed the conditioned contextual fear responses effects [49]. Allopregnanolone might be directly interacting with the GABA_A_ receptor at neurosteroid recognition sites (e.g., the α4 and α6 subunits) [50], [51] leading to potent anxiolytic and anticonvulsant properties and might impair PTSD-induced memory performance [1]. In our experiment, the anti-PTSD effects of midazolam were reversed by finasteride, an inhibitor of steroidogenic enzymes which reduced the production of allopregnanolone [25], [26]. This result agreed with the aforementioned studies. These actions may represent the underlying mechanism of the anti-PTSD effects of midazolam.

On the other hand, as a typical benzodiazepines drug, midazolam was known as enhancing GABA_A_ receptor inhibition by direct actions on central benzodiazepines receptor. Therefore a specific antagonist of the central benzodiazepines receptor, flumazenil [28], was used to exam whether this receptor was involved in the anti-PTSD effects induced by midazolam. Our present study demonstrated that flumazenil reversed the anti-PTSD effects of midazolam. This result was consistent with previous report that through both central benzodiazepine receptor and TSPO activation, midazolam inhibited hipocampal long-term potentiation [25].

## Conclusion

The present study demonstrated that repeated administration of midazolam ameliorated behavioral deficits in the SPS model without affecting spontaneous motor activity. The anti-PTSD effects of midazolam were mediated at least partially by TSPO and CBR and neurosteroidogenesis.
