# Correction: Midazolam Ameliorates the Behavior Deficits of a Rat Posttraumatic Stress Disorder Model through Dual 18 kDa Translocator Protein and Central Benzodiazepine Receptor and Neurosteroidogenesis

**DOI:** 10.1371/journal.pone.0119037

**Published:** 2015-03-05

**Authors:** The PLOS ONE Staff

The administration method of midazolam, flumazenil, and finasteride appears incorrectly as “p.o.” throughout the article. The correct administration method is “i.p.”
